# Immunohistochemistry as a tool for identifying *EGFR* amplification in CNS tumors

**DOI:** 10.1111/bpa.70073

**Published:** 2026-01-28

**Authors:** Arnault Tauziède‐Espariat, Mary Bah, Farah Sassi, Noémie Pucelle, Charlotte Berthaud, Marion Mandoula, Zeina Dababou, Noah Carnes, Leïla Brissez, Lauren Hasty, Euphrasie Servant, Alice Métais, Pascale Varlet

**Affiliations:** ^1^ Department of Neuropathology GHU Paris ‐ Psychiatry and Neuroscience, Sainte‐Anne Hospital Paris France; ^2^ Institut de Psychiatrie et Neurosciences de Paris (IPNP) UMR S1266, INSERM, IMA‐BRAIN Paris France; ^3^ Université de Paris Paris France

**Keywords:** amplification, EGFR, glioblastoma, immunohistochemistry

## Abstract

*EGFR* gene amplification constitutes a diagnostic hallmark for glioblastoma, IDH‐wildtype (GB, IDH‐WT). Herein, we demonstrated that EGFR IHC is a highly specific and sensitive biomarker for identifying *EGFR* amplification and should be part of the neuropathologist's routine panel of antibodies.

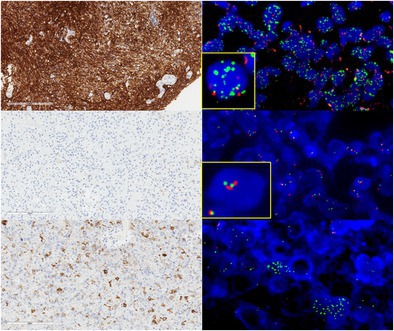

*EGFR* gene amplification constitutes a diagnostic hallmark for glioblastoma, IDH‐wildtype (GB, IDH‐WT), occurring in 40% of cases. It has also been reported in diffuse midline gliomas (DMG), H3K27‐altered, subtype *EGFR‐*altered [1], and has been consequently defined as one of the three molecular surrogate biomarkers in the World Health Organization's (WHO) 2021 Classification of Central Nervous System (CNS) tumors and in the recent update 11 of the cIMPACT‐NOW [2, 3]. Several methods are routinely used to identify this alteration: fluorescent in situ hybridization (FISH), next‐generation sequencing (NGS), CGH‐ and SNP‐array, and, more recently, copy number variation (CNV) from DNA‐methylation analysis. For rapid patient treatment in an increasingly complex diagnostic spectrum, neuropathologists may have easy routine diagnostic tools to confirm a diagnosis of GB, IDH‐WT. In this context, we evaluated the sensitivity and specificity of immunohistochemistry (IHC) for the detection of an *EGFR* amplification in a large cohort of CNS samples where the EGFR status was known.

We studied a cohort of 200 CNS samples including: 100 GB, IDH‐WT, 22 diffuse pediatric‐type high‐grade gliomas, H3‐ and IDH‐wildtype (pedHGG, H3‐ and IDH‐WT), 13 DMG, H3K27‐altered, 13 astrocytomas, IDH‐mutant, 11 gliomas, not elsewhere classified (NEC), 7 gangliogliomas, 7 gliosis, 6 pilocytic astrocytomas, 6 high‐grade astrocytomas with piloid features (HGAP), 3 diffuse hemispheric gliomas, H3 G34‐mutant, 2 oligodendrogliomas, IDH‐mutant and 1p/19q‐codeleted, 2 diffuse astrocytomas, *MYBL1*‐altered, 2 pleomorphic xanthoastrocytoma (PXA), 2 infant‐type hemispheric gliomas, 1 CNS tumor with *EP300::BCOR* fusion, 1 neuroepithelial tumor, *PATZ1*‐fused, 1 CNS neuroblastoma, *FOXR2‐*activated and 1 diffuse glioneuronal tumor with oligodendroglioma‐like features and nuclear clusters. All the tumor diagnoses were DNA‐methylation confirmed.

We used the EGFR antibody (monoclonal, clone 113; 1:200 dilution; Leica Biosystems; Deer Park, USA), targeting the extracellular domain of the receptor, on 3 μm‐thick sections of formalin‐fixed, paraffin‐embedded tissue samples, performed by an Omnis automate. Tumoral molecular analysis of *EGFR* was conducted using FISH and CNV from DNA‐methylation profiling analyses. Amplification was defined as more than 8 copies of the locus in at least 10% of tumor cells for FISH, as previously reported [4] and/or a log rank ratio >0.8 on CNV analysis. EGFR IHC was quantified using two methods based on the intensity of the staining (0: absent; 1: weak; 2: moderate; 3: strong) and the percentage of immunopositive tumor cells. A cytoplasmic and/or membranous staining was considered. Positivity was defined as 1/the product score being >200 as previously published [5], or 2/ if at least 10% of the tumor cells presented a strong immunoreactivity in correlation with the cut‐off used for the amplification using FISH. In case of discrepancy between IHC and CNV/FISH results, an NGS analysis to look for *EGFR* mutations was performed.

An *EGFR* amplification was present in 55 samples (33 GB, IDH‐WT, 10 adult‐type diffuse glioma without microvascular proliferation or necrosis requiring molecular analyses, 10 diagnoses hesitating between GB, IDH‐WT, and PXA, 1 pediatric‐type diffuse high‐grade glioma, and 1 diffuse midline glioma, H3K27‐altered). A positive IHC score was present in 44 (22%) and 57 samples (28%) respectively, using scoring Methods 1 and 2, respectively, with 41 (93%) and 55 (96%) of them harboring an *EGFR* amplification (Figure 1 and Table S1). All positive samples presented both cytoplasmic and membranous staining. IHC results were positive with both methods in 41 samples and negative with both methods in 140 cases, in line with CNV/FISH results. For 19 samples, IHC results were discordant between the two methods: Method 2 was positive while Method 1 was negative (*n* = 14), highlighting the fact that 10% of tumor cells with strong staining (positive result for Method 2 but negative for Method 1) was sufficient to detect amplification which was present in these 14 cases. Moreover, an IHC result was positive with Method 1 (*n* = 3), despite the absence of strong staining, but was not correlated with the presence of amplification. Thus, the sensitivity and specificity of EGFR IHC for the detection of *EGFR* amplification was 75% and 98% using Method 1, and 100% and 99% using Method 2. Using Method 2, two cases were considered as immunopositive and presented a concomitant gain (up to 6 copies of the locus) and mutation of *EGFR* (Figure 2). The integrated diagnoses of these two cases were GB, IDH‐WT, and a pedHGG, H3‐ and IDH‐WT. In 5/55 (9%) tumors with *EGFR* amplification, the alteration was subclonal and the IHC was positive only in the amplified component (Figure S1). Interestingly, the presence of an EGFR overexpression (using Method 2) and an amplification was present in 23 samples with diagnostic difficulties: 10 adult‐type diffuse glioma without microvascular proliferation or necrosis, 10 GB, IDH‐WT versus PXA, and 3 GB, IDH‐WT in patients aged <55 years‐old. Results for *EGFR* using FISH and CNV were concordant except in one case, where the amplification was subclonal on FISH and not detected on the CNV.

**FIGURE 1:**
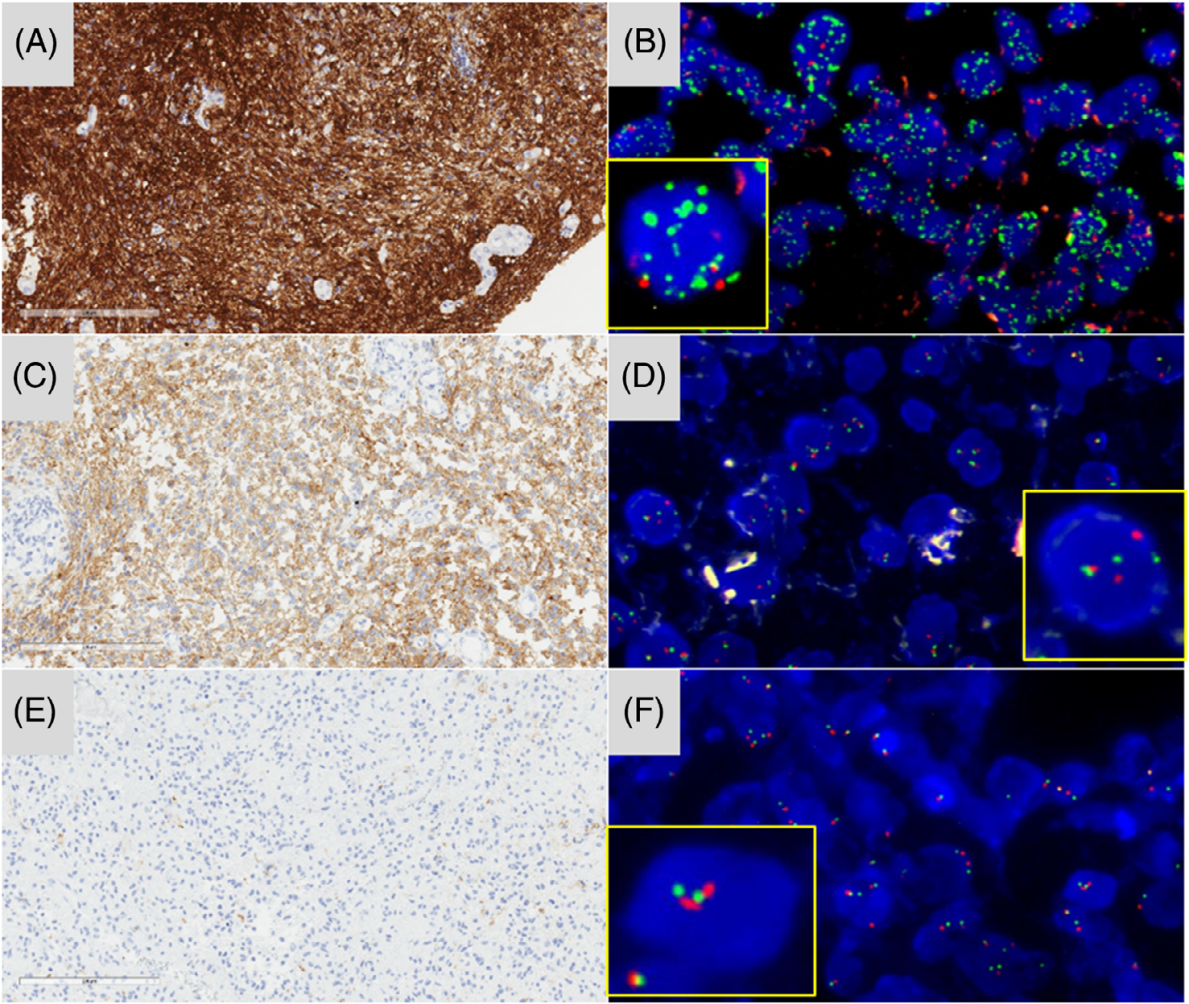
*EGFR* immunostaining and correlation with FISH analysis. Diffuse and strong staining for EGFR (A, magnification ×200), correlated with diffuse amplification of the locus *EGFR* (B, EGFR: Green signals; centromere of chromosome 7: Orange signals, magnification ×600). A diffuse but moderate staining for EGFR (C, magnification ×200), correlated with the gain of the *EGFR* locus in a context of polysomy (D, EGFR: Green signals; centromere of chromosome 7: Orange signals, magnification ×600). No staining for EGFR (E, magnification ×200), correlated with disomy of the *EGFR* locus (F, EGFR: Green signals; centromere of chromosome 7: Orange signals, magnification ×600). Scale bars represent 200 μm.

**FIGURE 2:**
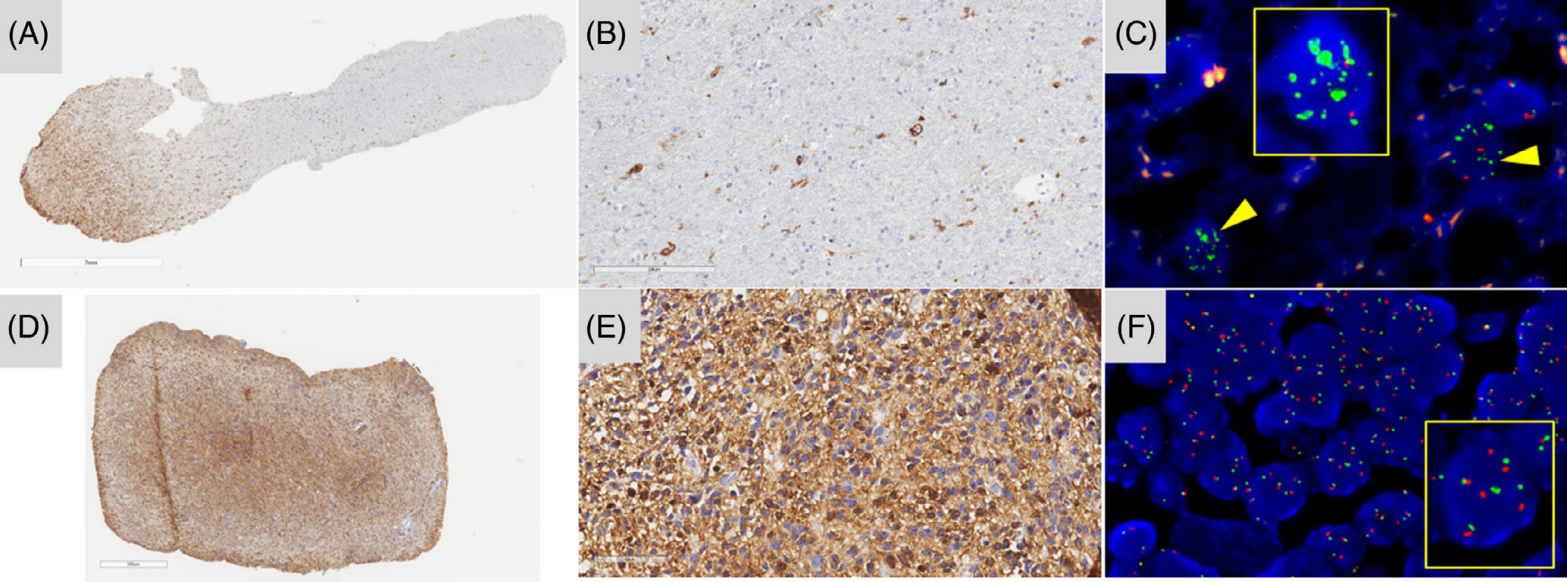
*EGFR* immunostaining and correlation with FISH analysis in discrepant or difficult cases. The case of a biopsy with a variation of cellularity: At left, a high number of EGFR immunopositive tumor cells and at right, a few tumor cells infiltrating the brain parenchyma (A, magnification, 4× and B, magnification ×200), correlated with an amplification of the *EGFR* locus in rare tumor cells (C, EGFR: Green signals; centromere of chromosome 7: Orange signals, magnification ×600). Diffuse and strong staining for EGFR (D, magnification, 4x and E, magnification, ×200), without amplification of the *EGFR* locus but with a high polysomy of up to six copies of the gene (F, EGFR: Green signals; centromere of chromosome 7: Orange signals, magnification ×600). Scale bars represent 2 mm (A, D), 200 μm (B, E), and 50 μm (C, F).

Altogether, EGFR IHC constitutes a fast, low‐cost, and conservative tissue‐consuming method to detect *EGFR* amplification. In adults, the diagnosis of GB, IDH‐WT can be challenging because of sampling (biopsies made outside the tumor enhancement or biopsies presenting low cellular rates), but also because of several histopathological pitfalls, some of which were recently described. Among them, we find the high‐grade glioma with pleomorphic and pseudopapillary features, HGAP, PXA, CNS tumors with *EP300::BCOR* fusion, glioblastoma with a primitive neuronal component, and high‐grade glioma subtype F; none of which have been reported to harbor an *EGFR* amplification. The presence of an *EGFR* amplification may also help to differentiate ganglioglioma/polymorphous low‐grade neuroepithelial tumor of the young from GB, IDH‐WT in the *FGFR3::TACC3‐*fused spectrum [6]. Nevertheless, the presence of the amplification may be encountered in other diffuse gliomas, more classically observed in children/adolescents and exceptionally observed in adults [7], such as DMG, *EGFR‐*altered [1], pedHGG, H3‐ and IDH‐WT, particularly subtypes RTK2A/B [8]. In these conditions, IHC constitutes a robust technique to confirm the diagnosis of GB, IDH‐WT, and to rule out differential diagnoses. Moreover, IHC may help to diagnose, alongside FISH analysis, the presence of an intratumoral heterogeneity or the presence of a subclonal amplification. Currently, IHC for EGFR is not recommended to detect the amplification of the gene by the cIMPACT‐NOW Update 3 [9], based on one previous study using another anti‐EGFR antibody than its used here [10]. However, as previously reported [11], we showed that a well‐calibrated EGFR IHC (with monitoring of positive and negative controls) may help to identify an *EGFR* amplification. The scoring, based only on strong immunoreactivity (Method 2), membranous and/or cytoplasmic, seems to be the more predictive method for the identification of the amplification because the other method may qualify a case as positive if 100% of tumor cells present moderate staining, but finally does not correlate with the presence of an amplification (but only a gain). This new scoring method needs to have well‐defined negative and positive controls for each staining score, as is routinely done for HER2 staining.

To conclude, we demonstrated that EGFR IHC is a highly specific and sensitive biomarker for identifying *EGFR* amplification and should be part of the neuropathologist's routine panel of antibodies. In the future, this IHC may also potentially help neurooncologists include patients in new clinical trial and treatment regimens [12].

## AUTHOR CONTRIBUTIONS

ATE and MB conducted the interpretation of the immunohistochemical data analysis and drafted the manuscript. ATE and MB generated the cohort, AM, LH, ES, FS, and PV helped in the revision of the manuscript. LB, CB, NP, MM, ZD, and NC conducted the immunohistochemical techniques. All authors reviewed the article.

## CONFLICT OF INTEREST STATEMENT

The authors declare no conflict of interest.

## Supporting information


**Supplementary Figure S1.** Intra‐tumoral heterogeneity of EGFR immunostaining and correlation with FISH analysis. Example of a case with intratumoral heterogeneity and no immunopositive tumor cells at left correlated with an EGFR locus disomy in the left part of the tumor (A, magnification, ×200, and B, C magnification ×400), and strong isolated tumor cells showing a subclonal amplification at right (D, E, EGFR: green signals; centromere of chromosome 7: orange signals, magnification ×600). Scale bars represent 300 μm (A), 200 μm (B–D), and 50 μm (C–E).

**Supplementary Table S1.** Detailed histopathological and molecular results of the cohort.

## Data Availability

The data that support the findings of this study are available from the corresponding author upon reasonable request.

## References

[1] Sievers P , Sill M , Schrimpf D , Stichel D , Reuss DE , Sturm D , et al. A subset of pediatric‐type thalamic gliomas share a distinct DNA methylation profile, H3K27me3 loss and frequent alteration of EGFR. Neuro‐Oncol. 2021;23(1):34–43. 33130881 10.1093/neuonc/noaa251 PMC7850075

[2] Louis DN , Perry A , Wesseling P , Brat DJ , Cree IA , Figarella‐Branger D , et al. The 2021 WHO classification of tumors of the central nervous system: a summary. Neuro‐Oncol. 2021;23(8):1231–1251. 34185076 10.1093/neuonc/noab106 PMC8328013

[3] Wesseling P , Capper D , Reifenberger G , Sarkar C , Hawkins C , Perry A , et al. cIMPACT‐NOW update 11: proposal on adaptation of diagnostic criteria for IDH‐ and H3‐wildtype diffuse high‐grade gliomas and for posterior fossa ependymal tumors. Brain Pathol. 2025;35:e70035. 10.1111/bpa.70035 PMC12695693 40887057

[4] Tauziède‐Espariat A , Saffroy R , Pagès M , Pallud J , Legrand L , Besnard A , et al. Cerebellar high‐grade gliomas do not present the same molecular alterations as supratentorial high‐grade gliomas and may show histone H3 gene mutations. Clin Neuropathol. 2018;37(5):209–216. 29809131 10.5414/NP301104

[5] Pirker R , Pereira JR , von Pawel J , Krzakowski M , Ramlau R , Park K , et al. EGFR expression as a predictor of survival for first‐line chemotherapy plus cetuximab in patients with advanced non‐small‐cell lung cancer: analysis of data from the phase 3 FLEX study. Lancet Oncol. 2012;13(1):33–42. 22056021 10.1016/S1470-2045(11)70318-7

[6] Wu Z , Lopes Abath Neto O , Bale TA , Benhamida J , Mata D , Turakulov R , et al. DNA methylation analysis of glioblastomas harboring FGFR3‐TACC3 fusions identifies a methylation subclass with better patient survival. Acta Neuropathol. 2022;144(1):155–157. 35567606 10.1007/s00401-022-02430-7 PMC10572100

[7] Paoli C , Mc Leer A , Boyer J , Mondot L , Dadone‐Montaudié B , Godfraind C , et al. A 78‐year‐old woman with diffuse white matter infiltration and predominant involvement of bilateral temporo‐parieto‐occipital regions. J Cell Mol Med. 2024;28(8):e18245. 38613356 10.1111/jcmm.18245 PMC11015387

[8] Tauziède‐Espariat A , Friker LL , Nussbaumer G , Bison B , Dangouloff‐Ros V , Métais A , et al. Diffuse pediatric high‐grade glioma of methylation‐based RTK2A and RTK2B subclasses present distinct radiological and histomolecular features including Gliomatosis cerebri phenotype. Acta Neuropathol Commun. 2024;12(1):176. 39558399 10.1186/s40478-024-01881-1 PMC11575044

[9] Brat DJ , Aldape K , Colman H , Holland EC , Louis DN , Jenkins RB , et al. cIMPACT‐NOW update 3: recommended diagnostic criteria for «diffuse astrocytic glioma, IDH‐wildtype, with molecular features of glioblastoma, WHO grade IV». Acta Neuropathol. 2018;136(5):805–810. 30259105 10.1007/s00401-018-1913-0 PMC6204285

[10] Lee M , Kang SY , Suh YL . Genetic alterations of epidermal growth factor receptor in glioblastoma: the usefulness of immunohistochemistry. Appl Immunohistochem Mol Morphol. 2019;27(8):589–598. 29912767 10.1097/PAI.0000000000000669

[11] Burel‐Vandenbos F , Turchi L , Benchetrit M , Fontas E , Pedeutour Z , Rigau V , et al. Cells with intense EGFR staining and a high nuclear to cytoplasmic ratio are specific for infiltrative glioma: a useful marker in neuropathological practice. Neuro‐Oncol. 2013;15(10):1278–1288. 23935154 10.1093/neuonc/not094 PMC3779042

[12] Friedman JS , Jun T , Rashidipour O , Huang KL , Ellis E , Kadaba P , et al. Using EGFR amplification to stratify recurrent glioblastoma treated with immune checkpoint inhibitors. Cancer Immunol Immunother CII. 2023;72(6):1893–1901. 36707424 10.1007/s00262-023-03381-y PMC10992363

