# Supplementary figures and images for: Immunohistochemistry as a tool for identifying EGFR amplification in CNS tumors
Source: Brain Pathol. 2026 Jan 28;36(4):e70073. doi: 10.1111/bpa.70073 (PMC13239168; doi:10.1111/bpa.70073)

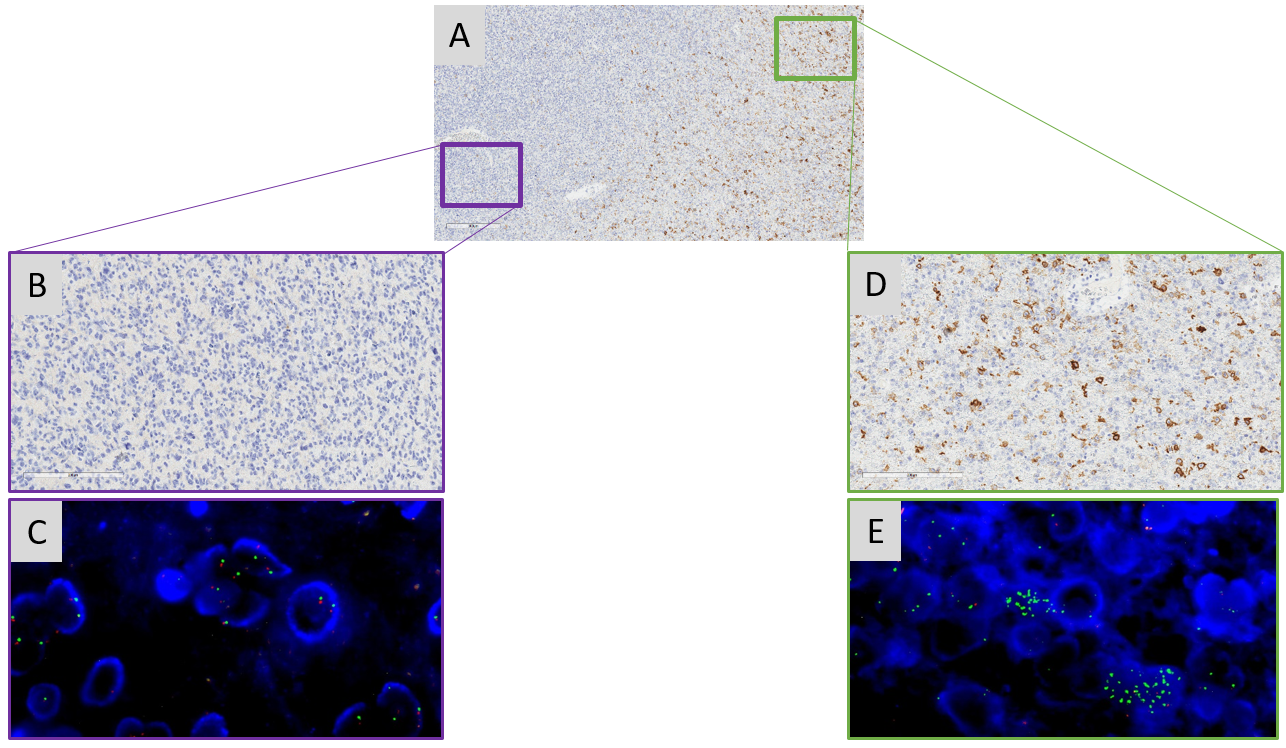

Supplement: Supplementary file 1 — Supplementary Figure S1. Intra‐tumoral heterogeneity of EGFR immunostaining and correlation with FISH analysis. Example of a case with intratumoral heterogeneity and no immunopositive tumor cells at left correlated with an EGFR locus disomy in the left part of the tumor (A, magnification, ×200, and B, C magnification ×400), and strong isolated tumor cells showing a subclonal amplification at right (D, E, EGFR: green signals; centromere of chromosome 7: orange signals, magnification ×600). Scale bars represent 300 μm (A), 200 μm (B–D), and 50 μm (C–E). [file BPA-36-e70073-s001.tif]
